# Toponymic knowledge infrastructures in five-state Middle Asia: linguistic transformation, manuscript cartography, and historical-geographical reconstruction

**DOI:** 10.3389/fsoc.2026.1876337

**Published:** 2026-10-14

**Authors:** Bibaisha Iliasovna Nurdauletova, Sholpan Saparbaikyzy, Zhambyl Omarovish Artykbaev, Tursynkhan Zakonuly Kairken, Saltanat Spatayeva, Elizabeth Shilunga

**Affiliations:** 1 Department of Kazakh Philology, Faculty of Philology and Sports, Sh. Yessenov Caspian University of Technologies and Engineering, Aktau, Kazakhstan; 2 Department of Pedagogy, Faculty of Pedagogy, Yessenov University, Aktau, Kazakhstan; 3 Department of Archaeology and Ethnology, L. N. Gumilyov Eurasian National University, Astana, Kazakhstan; 4 Department of Oriental Studies, L. N. Gumilyov Eurasian National University, Astana, Kazakhstan; 5 Department of Kazakh Linguistics, Faculty of Philology, L. N. Gumilyov Eurasian National University, Astana, Kazakhstan; 6 Department of Environmental Engineering and Management, The University of Dodoma, Dodoma, Tanzania

**Keywords:** cross-script harmonisation, geolinguistic reconstruction, historical toponymy, Kazakhstan, manuscript knowledge systems, Middle Asia

## Abstract

Toponymic records embedded in Arabic, Persian, and Chinese manuscript traditions provide evidence for reconstructing the historical geographies of Kazakhstan, Kyrgyzstan, Tajikistan, Turkmenistan, and Uzbekistan. This review uses a structured narrative and conceptual-synthesis methodology to compare place-name transmission across languages, scripts, manuscript genres, and political regimes. A diachronic framework separates pre-Islamic external attestations, medieval Arabic-Persian documentation, early-modern cartography, imperial and Soviet standardisation, and post-Soviet renaming rather than treating linguistic layers as synchronous. Direct evidence from the five-state core is distinguished from supporting evidence from neighbouring transmission corridors and comparative regions. Micro-cases of Koneurgenish/Gurganj, Dargan/Dargan-Ata, hydronymic compounds, and political renaming are combined with explicit cross-script harmonisation rules and spatial-chronological triangulation. Toponymy is operationalised as a sociopolitical knowledge infrastructure when names function as repeatable identifiers linking routes, jurisdictions, administrative records, gazetteers, and maps. Digital approaches extend this framework through georeferenced historical gazetteers, variant-name alignment, route modelling, and confidence-ranked reconstruction. Reliable interpretation depends on preserving source provenance, separating transliteration from phonological reconstruction, and distinguishing linguistic change from administrative replacement.

## 1 Introduction

Toponymy provides a durable archive for reconstructing historical geography where documentary records are discontinuous, multilingual, or politically mediated. In the five-state Middle Asian core, place names preserve linguistic and spatial evidence from the Kazakh steppe, the Kyrgyz and Tajik highlands, the Bukhara-Samarkand and Khorezm oases, and the Amu Darya and Syr Darya corridors. Recent research on ancient Turkic toponyms of Kazakhstan shows that geographical names encode historical realities, social organisation, economic activities, and environmental knowledge (Imanberdiyeva et al., 2025). Work in North Kazakhstan likewise documents recurring lexical relationships among settlement, pasture, water, and ethnonymic naming (Sabieva and Kakimova, 2025). These regional records accumulated under successive patterns of mobility, state formation, and transregional exchange (Paik and Shahi, 2023). Their evidentiary value increases when linguistic interpretation is tested against route geography, political context, and the chronology of written attestation (Williamson, 2023). Comparative studies are retained where they clarify transferable questions of place-name persistence, spatial interpretation, naming authority, and cultural memory (Valkó et al., 2023; Kersapati, 2023; El Shakry, 2021; Mohammadpour and Soleimani, 2022; Hakala et al., 2015; Lim Tyan Gin and Perono Cacciafoco, 2021; Yulifar et al., 2023), rather than as substitutes for direct Middle Asian evidence.

The Silk Road intensified cross-script transmission because the five-state core was linked to itineraries, trading nodes, frontier administrations, and manuscript centres extending eastward to the Tarim Basin and Dunhuang and westward toward Iranian and Islamic scholarly networks. Network reconstruction shows that Silk Road connectivity was distributed across multiple nodes and corridors (Shen et al., 2024). Dunhuang manuscript scholarship provides an East Asian control for examining how multilingual documentary archives preserve changing geographic knowledge (Rong, 2025). Central Asian cartography provides a complementary western record: Kollmann’s analysis of Anthony Jenkinson’s sixteenth-century map documents the revision of cartographic schemes containing Tashkent and major river systems of the region (Kollmann, 2024). These neighbouring and transregional sources explain transmission into and across Kazakhstan, Kyrgyzstan, Tajikistan, Turkmenistan, and Uzbekistan while the five-state corpus remains the analytical centre.

Repeated migration, pastoral mobility, conquest, and administrative reorganisation produced overlapping naming layers within Middle Asia. Historical migration scholarship documents the long duration of demographic and political change in Central Asia (Stuti and Haq, 2025). The toponymic record makes parts of these transformations spatially visible. Kazakhstan studies identify Turkic place-name elements associated with environmental features, social organisation, and economic activity (Imanberdiyeva et al., 2025), while North Kazakhstan material preserves settlement names tied to seasonal pastoral practice and hydronyms tied to water characteristics (Sabieva and Kakimova, 2025). In oasis zones, Arabic and Persian manuscript traditions recorded urban centres, provincial terms, routes, and local variants through their own orthographic conventions. Place names are consequently treated as historically layered evidence whose interpretation requires comparison among language, script, geography, and institutional context. Related work on sacred landscapes, Turkic cultural memory, settlement history, and naming practice provides contextual support (Rosina and Maroufi, 2017; Shakirova et al., 2026; Sadirova et al., 2024; Pangereyev et al., 2023; Peng et al., 2020; Meiirbekov and Meirbekov, 2023).

Current scholarship has moved beyond purely etymological cataloguing toward data structures that connect name variants with time and space. Historical toponymic information systems demonstrate how place-name records can be organised as spatiotemporal data (Liu and Hu, 2017). Toponym-assisted georeferencing shows that historical place names can support map alignment when name evidence is linked to coordinates and uncertainty (Bahgat and Runfola, 2021). Work on historical travel records provides methods for extracting and relating place entities across textual sources (Ghosh and Chatterjee, 2025), while research on cartographic naming authority clarifies why map labels must also be interpreted as products of institutional power (Noucher, 2022). For Middle Asia, the unresolved problem is integration: direct regional attestations, historical-script variants, cartographic position, and provenance are still rarely evaluated within a single reproducible framework. Regional work on toponyms in folk prose provides an additional cultural-linguistic comparison (Shuriyeva et al., 2025).

This review synthesises historical geography, manuscript studies, philology, migration history, spatial data science, and digital cartography around four linked questions: how Arabic, Persian, and Chinese recording traditions encoded Middle Asian place names; how Turkic, Iranian, and hybrid strata can be differentiated within the five-state core; which mechanisms generated phonetic, semantic, political, and cartographic transformation; and which digital methods can integrate multilingual evidence without obscuring uncertainty. The analysis treats individual toponyms as source-critical observations whose value depends on provenance, chronology, spatial plausibility, and cross-source agreement. A diachronic principle governs all four questions: a linguistic layer is interpreted through dated attestations and institutional contexts rather than treated as a timeless stratum, and political replacement is analytically separated from regular phonological or morphological change.

The remainder of the review is organised as follows. Section 2 sets out the review design, search strategy, coding, quality appraisal, and uncertainty protocol. Section 3 compares Arabic, Persian, and Chinese recording systems. Section 4 examines Turkic, Iranian, and multilingual toponymic strata. Section 5 evaluates manuscript cartography and territorial visualisation. Section 6 classifies phonetic, semantic, and political transformations. Section 7 assesses ethnogenetic and environmental inferences from place-name evidence. Section 8 identifies the principal reconstruction bottlenecks. Section 9 develops the digital and computational infrastructure needed to address them. Section 10 synthesises the analytical implications and research trajectory.

## 2 Review methodology

### 2.1 Review design and rationale

An integrative structured narrative design combined evidence from historical manuscript studies, philology, cartography, critical toponymy, GIS, and computational geolinguistics. The design draws on source-critical approaches to naming practice and historical entity extraction (Völkel, 2023; Ghosh and Chatterjee, 2025) and on spatial methods that use place names as georeferencing evidence (Bahgat and Runfola, 2021). The object of analysis was the evidentiary movement of place names across languages, scripts, manuscript genres, and political regimes, with the empirical focus restricted to Kazakhstan, Kyrgyzstan, Tajikistan, Turkmenistan, and Uzbekistan. The methodological logic combined conceptual synthesis, comparative source criticism, and mechanism-based interpretation while separating primary evidence from the five-state core from contextual evidence drawn from neighbouring regions and wider Eurasian transmission systems. Methodological treatments that inform this design include (Choo, 2023; Tort-Donada and López-Leiva, 2023; Garcia-Quera, 2025; Perono Cacciafoco and Cavallaro, 2023; Tent, 2015).

### 2.2 Search strategy and source identification

Sources were identified through multidisciplinary scholarly databases, publisher platforms, archival bibliographies, citation chasing, and targeted searches for manuscript, cartographic, linguistic, and digital-toponymy evidence. Regional source identification was anchored by studies directly addressing Kazakhstan, North Kazakhstan, the Bukhara oasis, and Central Asian historical cartography (Imanberdiyeva et al., 2025; Sabieva and Kakimova, 2025; Schwarz, 2022; Gorshenina, 2021). Search expressions combined regional, country-specific, historical, and source labels, including Middle Asia, Kazakhstan, Kyrgyzstan, Tajikistan, Turkmenistan, Uzbekistan, Kazakh steppe, Kyrgyz ranges, Tajik Pamirs, Turkmen oases, Uzbek oases, Transoxiana, Ferghana, Bukhara, Samarkand, Khorezm, Amu Darya, Syr Darya, Silk Road, Arabic geography, Persian historiography, Chinese gazetteers, manuscript cartography, historical toponymy, hydronymy, exonym, endonym, toponym matching, GIS, georeferencing, named-entity recognition, and cross-script harmonisation. Supporting labels such as Tarim Basin, Xinjiang, Iranian plateau, Inner Asia, Afghanistan, Russia, China, and South Asia were used during exploratory searching when a source might explain transmission, comparison, or boundary mediation affecting the five-state core. South Asia was not retained as a distinct supporting evidence category in the final synthesis because no South Asia-centred source met the direct-attestation, transmission-corridor, or source-critical-method criteria specified in Section 2.9 without relying primarily on analogy. The final evidence base comprised 138 unique references after relevance screening and de-duplication. English-language scholarship was prioritised, with direct evidence from the five-state core and neighbouring East Asian source traditions given greater interpretive weight than geographically distant analogies. Because the search combined database discovery, publisher platforms, archival bibliographies, citation chasing, and targeted source identification, it is reported as a structured narrative synthesis rather than a systematic review; database-specific hit counts and a PRISMA-style screening flow are not asserted where they were not prospectively recorded.

### 2.3 Eligibility and exclusion criteria

Included sources met at least one of five criteria: direct relevance to toponymic evidence from Kazakhstan, Kyrgyzstan, Tajikistan, Turkmenistan, or Uzbekistan; documentation of Arabic, Persian, Chinese, Turkic, Iranian, or hybrid naming evidence affecting the five-state core; explanation of a linguistic, cartographic, administrative, or political transformation mechanism; contribution to manuscript cartography, historical gazetteer interpretation, or route reconstruction for Middle Asian routes, oases, steppe corridors, and frontier interfaces; or provision of computational, GIS, or database methods applicable to toponym reconstruction. Direct regional examples include ancient Turkic and North Kazakhstan toponymy and water-related settlement names in the Manas River Basin (Imanberdiyeva et al., 2025; Sabieva and Kakimova, 2025; Zhang et al., 2025). Sources centred on neighbouring or distant regions entered the corpus only when they supplied comparative mechanisms, transmission evidence, methodological controls, or contextual framing. Exclusion criteria covered local naming inventories without reconstructive relevance, sources with unclear provenance, unsupported nationalist or essentialist claims, works that interpreted modern administrative spellings as direct evidence of ancient usage, and materials whose claims lacked linguistic, spatial, or historical support. Primary-source status was reserved for directly reproduced or directly cited manuscript and cartographic materials. Historical attestations available only through scholarly editions or source-critical studies were treated as mediated evidence and were not represented as independently verified primary readings.

### 2.4 Data extraction and analytical coding

For each source, evidence was extracted into analytical categories: country or subregion, language or script tradition, toponym type, geographic referent, manuscript or archival context, date of attestation, proposed historical layer, transformation mechanism, political-institutional setting, spatial reliability, and uncertainty. The transformation coding follows empirically documented processes such as transliteration, phonetic adjustment, calquing, reinterpretation, and renaming (Zharkynbekova et al., 2024) while the political reading of map labels is treated as a separate source-critical dimension (Noucher, 2022). Each item was coded as primary evidence when it concerned Kazakhstan, Kyrgyzstan, Tajikistan, Turkmenistan, or Uzbekistan and as supporting evidence when it concerned neighbouring regions that supplied comparative, transmission, or methodological framing. Toponyms were classified as endonyms, exonyms, phonetic transcriptions, semantic translations, administrative standardisations, or political renamings without collapsing distinct variants into a single harmonised form. To enforce diachronic comparability, extraction also distinguished the political-administrative phase, the date and language of each attestation, the script in which a form is preserved, any explicitly reconstructed spoken form, morphological segmentation, semantic gloss, and the proposed direction of transmission. A phonetic value was recorded only when justified by the source or by a source-specific historical-phonological reconstruction; otherwise the analysis preserves transliteration without assigning an unsupported International Phonetic Alphabet value. This distinction prevents modern pronunciation from being projected backward onto medieval Arabic or Persian spellings or historical Chinese transcriptions.

### 2.5 Comparative synthesis procedure

Comparative synthesis followed four analytical stages. Arabic, Persian, and Chinese traditions were first analysed as recording systems that preserved or transformed place names associated with Kazakhstan, Kyrgyzstan, Tajikistan, Turkmenistan, and Uzbekistan. Turkic, Iranian, and hybrid strata were then compared across hydronyms, oronyms, settlement names, trade nodes, and administrative terms within the five-state core. Regional cross-script cases and modern matching methods provided controls for distinguishing written similarity from historically defensible equivalence (Schwarz, 2022; Zharkynbekova et al., 2024; Qiu et al., 2024). Transformation mechanisms were subsequently classified as phonetic, semantic, political, cartographic, or administrative, and the reconstructed evidence was finally linked to methodological bottlenecks and digital infrastructures. This sequence maintained a mechanism-centred and geographically bounded synthesis.

### 2.6 Quality appraisal and triangulation

Source reliability was assessed through provenance, disciplinary quality, evidentiary specificity, chronological clarity, and consistency with independently attested geography. Evidence from Kazakhstan, Kyrgyzstan, Tajikistan, Turkmenistan, and Uzbekistan carried primary interpretive weight, while sources from neighbouring regions tested mechanisms, clarified transmission, or contextualised frontier mediation. No single attestation carried decisive force. Reconstruction confidence increased where several controls converged, including phonological plausibility, route position, hydrological or topographic continuity, administrative context, manuscript genealogy, archaeological correlation, or cross-linguistic parallel forms. Source-critical work in the Bukhara region and Central Asian historical cartography illustrates the value of combining textual form with relative geography (Schwarz, 2022; Gorshenina, 2021). Conflicting forms were retained as variants where the evidence did not justify harmonisation.

### 2.7 Uncertainty management

Uncertainty was handled explicitly because toponymic evidence often separates original naming from first written attestation. Chronological coding distinguished the date of name origin, manuscript recording, later copying, and administrative standardisation. It also separated linguistic similarity from identity, spatial proximity from equivalence, political visibility from community usage, and external attestation from internal geographic evidence within the five-state core. Bukhara source criticism demonstrates the need to preserve competing identifications until relative geography and parallel forms converge (Schwarz, 2022), while Kazakhstan transliteration research shows why several script forms can legitimately coexist for one referent (Zharkynbekova et al., 2024). Where reconstruction remained uncertain, the interpretation was reported as a ranked confidence assessment rather than a definitive identification.

### 2.8 Terminology and geographic scope

Middle Asia denotes the five-state region comprising Kazakhstan, Kyrgyzstan, Tajikistan, Turkmenistan, and Uzbekistan. The geographic core comprises the Kazakh steppe, the Kyrgyz and Tajik highland interfaces, the Ferghana Valley, the Bukhara-Samarkand and Khorezm oasis systems, the Amu Darya and Syr Darya corridors, Transoxiana/Mawarannahr, and Silk Road nodes located in or directly connected to these five states. The historical-geographical framing is consistent with scholarship on the region’s position between Iranian, Inner Asian, and wider Eurasian spatial imaginaries (Wastnidge et al., 2017). The term is used as a bounded category for manuscript circulation, trade connectivity, steppe-oasis interaction, and frontier mediation.

### 2.9 Treatment of supporting regions

Evidence from the Tarim Basin, Xinjiang, the Iranian plateau, Inner Asia, Afghanistan, Russia, China, and wider Eurasian corridors provides contextual support for toponymic transmission into and across Kazakhstan, Kyrgyzstan, Tajikistan, Turkmenistan, and Uzbekistan. Dunhuang manuscript research and Xinjiang toponymic-landscape research are included as neighbouring eastern evidence (Rong, 2025; Guo et al., 2025), and Chinese toponym matching is used as a methodological control for logographic variant resolution (Qiu et al., 2024). These neighbouring zones remain secondary to the five-state corpus and are used to clarify manuscript transmission, exonyms, route knowledge, administrative mediation, and cross-script distortion.

Supporting-region evidence was retained only when it satisfied at least one of three relevance tests: it provided an external attestation of a place in the five-state core; it documented a transmission corridor through which a core toponym moved between linguistic or manuscript systems; or it supplied a methodologically comparable script or administrative mechanism needed to evaluate a core reconstruction. This rule prevents peripheral material from enlarging the geographical object of study by analogy alone. The Iranian plateau is used principally to clarify Persianate mediation, whereas the Tarim Basin, Xinjiang, Dunhuang, and Chinese records are used as eastern transmission or external-attestation controls (Wastnidge et al., 2017; Rong, 2025; Guo et al., 2025). Modern Chinese matching studies enter only as methodological controls for logographic variant resolution rather than as evidence about Middle Asian historical forms (Qiu et al., 2024). South Asia was examined during exploratory searching but is not treated as a separate supporting region in the final synthesis because no South Asia-centred source in the screened corpus satisfied these tests strongly enough to support a five-state reconstruction without analogy. Evidence without a demonstrable transmission, attestation, or methodological link to Kazakhstan, Kyrgyzstan, Tajikistan, Turkmenistan, or Uzbekistan was excluded from interpretive claims. No peripheral case is used by itself to establish the origin, chronology, or linguistic lineage of a place in the five-state core; such claims require direct core-region evidence or convergent evidence tied to an identifiable transmission pathway.

### 2.10 Methodological limitations

Limitations arise from the uneven survival of manuscripts, the selective interests of imperial record keepers, the instability of cross-script transcription, and the absence of complete georeferenced corpora for many historical place names in Kazakhstan, Kyrgyzstan, Tajikistan, Turkmenistan, and Uzbekistan. Historical travel-record extraction and regional digital-atlas work show both the potential and the present incompleteness of integrated infrastructures (Ghosh and Chatterjee, 2025; Nazarkulova and Strobl, 2015). The evidence base is not exhaustive across every toponym, manuscript tradition, or neighbouring region. The methodological synthesis therefore provides a bounded framework for evaluating how names moved across languages, scripts, political regimes, and spatial infrastructures. Because source density differs among the five states, country-level claims are made only where direct evidence exists; region-wide statements are reserved for mechanisms supported by multiple sources or shared transregional systems. Evidence-rich cases are not automatically generalised across Kazakhstan, Kyrgyzstan, Tajikistan, Turkmenistan, and Uzbekistan.

## 3 Civilizational knowledge systems and the recording of space

### 3.1 Arabic geographical epistemology

Arabic geographical writing recorded Middle Asian space through routes, urban sequences, commercial connections, pilgrimage, and regional description. In the five-state core, Bukhara, Samarkand, Khorezm, and the Transoxianan corridors entered manuscript traditions in which practical sequence and connectivity could be more important than metric distance. Scholarship on Arabic-Islamic geography demonstrates the coexistence of mathematical, descriptive, and itinerary-based modes (Ducène, 2021) and the long history of Arabic geographic interpretation of Inner Asian space (Tolmacheva, 1995). These records preserve repeated attestations, but Arabic orthography does not encode every vowel contrast required for Turkic reconstruction. Source criticism in the Bukhara region shows why written form must be tested against relative geography before two historical labels are treated as the same place (Schwarz, 2022). Arabic attestations are therefore evaluated as institutionally produced records with identifiable orthographic and spatial constraints. Comparative studies of manuscript transmission, Islamic cartography, codicology, and phonetic transfer define the controls used to interpret copied and translated names (Morton, 2019; Yahya and Jones, 2021; Cruse, 2017; Apellániz, 2020; Hofer, 2020; Flege and Port, 1981; Ahn and Juraev, 2025). Work on Ottoman and Arabic-Islamic geographic representation further clarifies how cartographic form and linguistic structure shape recorded place names (Soucek, 2011; Travassos and Amorim Filho, 2016; Bobeck, 2025).

### 3.2 Persian historiographical mediation

Persian historiographical and administrative writing provided a second major channel for stabilising names of oasis centres, dynastic territories, fiscal districts, and routes. In the Bukhara-Samarkand and Khorezm zones, Persian literary and bureaucratic practice interacted continuously with Turkic naming, producing transliterations, Persianised forms, and hybrid names. Within the Bukhara oasis, proposed identifications of peripheral settlements require comparison of manuscript variants with relative location, neighbouring place sequences, and administrative context (Schwarz, 2022). This source-critical procedure is especially important where several spellings could plausibly refer to the same locality. The Persian record is strongest when manuscript genealogy, relative geography, administrative context, and local linguistic evidence converge; an isolated resemblance in spelling is insufficient for identification.

### 3.3 Chinese dynastic Toponymic encoding

Chinese dynastic and gazetteer traditions provide an external eastern witness to routes, polities, and place names associated with the Middle Asian frontier. Their evidentiary value is especially important for chronological comparison because foreign names were repeatedly rendered through Chinese characters whose historical pronunciations and graphic conventions changed over time. A Bukhara-region example is the Chinese form Niumi discussed in source-critical work on settlements behind Bukhara; its identification depends on relative geography and parallel forms as well as phonetic resemblance (Schwarz, 2022). Modern Chinese toponym-matching and historical named-entity recognition research demonstrates the computational difficulty of linking variant forms across texts (Qiu et al., 2024; Liu et al., 2021), while gazetteer information systems show how names can be joined to temporal and spatial metadata (Hung et al., 2008). These methods support reconstruction of Middle Asian records while modern Chinese case studies remain methodological controls rather than substitutes for evidence from the five-state core. East Asian studies of naming politics, settlement toponymy, administrative geography, and historical regional relations provide contextual controls (Wu and Young, 2023; Qian et al., 2016; Chan, 2016; Liu et al., 2024b; Jiao and Lu, 2024; Wang et al., 2019; Zhao et al., 2020). Historical river reconstruction, annotated corpora, Manchu-language evidence, inscriptional reform, and China-Central Eurasia studies provide complementary controls (Zhong et al., 2020; Ye et al., 2025; Zhu et al., 2018; Söderblom Saarela, 2020; Schneider, 2021).

### 3.4 Toponymy as a sociopolitical knowledge infrastructure

In this review, knowledge infrastructure denotes an institutionalised system in which place names operate as durable identifiers that allow spatial information to be recorded, copied, compared, retrieved, and acted upon across documents. The concept is operational rather than metaphorical. A toponym functions infrastructurally when it identifies a referent consistently enough for repeated use, links that referent to an administrative, route, jurisdictional, fiscal, or cartographic record, and is reproduced through an institution with authority to maintain, standardise, or replace the recorded form. Critical work on naming authority shows why official visibility is institutionally produced (Noucher, 2022), while gazetteer information systems demonstrate how names can become durable links among administrative, temporal, and spatial records (Hung et al., 2008).

The practical connection to statecraft is therefore testable. Arabic geographical traditions organised places through routes, urban sequences, and regional descriptions (Ducène, 2021), Persian historiographical and administrative writing stabilised oasis centres, territorial designations, and provincial relationships (Schwarz, 2022), and Chinese dynastic and gazetteer systems embedded place names within bureaucratically maintained spatial hierarchies (Hung et al., 2008). In these settings, a name could function as an index key connecting a settlement, route stage, river crossing, district, or mapped jurisdiction to other records. Renaming or orthographic standardisation consequently altered not only a label but also the identifier through which a place was retrieved and related to institutional knowledge. The framework does not assume that all five states shared one bureaucratic system; the infrastructural interpretation is applied only where provenance demonstrates institutional production, consistent with the broader relationship between mapping and territorial authority (Branch, 2011). Operationally, a case is treated as a sociopolitical knowledge infrastructure only when three components can be identified together: a named spatial referent, an institutional record or documentary genre that reproduces the name, and a governance function such as route management, jurisdictional or fiscal indexing, gazetteer standardisation, or cartographic assertion. This criterion separates infrastructural use from the mere survival of a place name in an isolated text. The qualitative descriptors used in Table 1 are comparative synthesis judgments derived from the cited literature; they are not measured or statistically calibrated scores.

**Table 1:** Comparative features of Arabic, Persian, and Chinese toponymic recording systems (see Sections 3.1–3.3).

Recording tradition	Recording medium	Phonetic fidelity	Administrative bias	Spatial precision	Temporal continuity	References
Arabic mathematical-geographic tradition	Translations and adaptations of Ptolemaic geography, astronomical tables, coordinate lists	Moderate. Consonantal structure preserves roots but omits short vowels, generating transliteration instability	Moderate. Often court-sponsored but not strictly bureaucratic in structure	Moderate to high. Incorporation of coordinate systems improved mathematical precision	High. Repeated adaptation and reuse across centuries	Ducène (2021); Tolmacheva (1995)
Arabic cultural-geographic manuscript tradition	Narrative geographies, regional descriptions, compiled geographic treatises	Moderate. Oral transmission and manuscript copying increased variant formation	Moderate. Reflects trade networks and scholarly centers more than rigid state bureaucracy	Schematic. Relative spatial ordering rather than coordinate mapping	High. Sustained manuscript copying traditions	Pinto (2016); Tolmacheva (1995)
Persian historiographical-administrative tradition	Court chronicles, provincial descriptions, fiscal or administrative records, and route narratives	Moderate. Turkic and local names may be Persianised or morphologically adapted	High in court and bureaucratic contexts; variable outside administrative centres	Moderate. Relative territorial organisation is often stronger than metric precision	High within textual traditions, with variation across dynastic and copying phases	Schwarz (2022)
Chinese dynastic administrative gazetteer system	Official gazetteers, dynastic histories, administrative atlases	Variable. Logographic transliteration approximates foreign phonemes with distortion	Very high. Central bureaucratic control structured naming and mapping	High within administrative jurisdictions	Very high. Continuous state-sponsored production across dynasties	Hu (2007); Hung et al. (2008)
Chinese modern local gazetteer system	Post-1949 local gazetteers, digitized gazetteer systems, metadata-integrated GIS platforms	Moderate to high within standardized Mandarin framework	Very high. Central regulatory guidelines govern compilation	High. Integrated spatial–temporal cataloguing and GIS linkage	Very high. Institutionalized, regulated continuation	Xue (2010)

## 4 Linguistic stratigraphy of five-state middle Asian Toponyms

This section examines the linguistic stratigraphy of the five-state core. Section 4.1 focuses on Turkic strata and diachronic persistence, Section 4.2 evaluates Iranian and hybridised layers, Section 4.3 considers multilingual convergence zones where several naming traditions refer to the same landscape, and Section 4.4 develops diachronic micro-cases and chronological sequencing.

### 4.1 Turkic strata and diachronic persistence

Turkic toponyms in the five-state Middle Asian core preserve recurring associations with mobility, land use, hydrology, settlement, and social memory. Kazakhstan provides some of the clearest documented examples in the present corpus. Ancient Turkic toponyms have been classified according to historical realities, social organisation, economic activity, and environmental knowledge (Imanberdiyeva et al., 2025). In North Kazakhstan, Kystaukaragai combines kystau, ‘winter quarters’, with karagai, ‘pine’, while Aul Zhailau draws on the term for summer pasture; the same corpus includes widespread hydronyms such as Aksu and Karasu and ethnonym-bearing names such as Kyrghyz upland and Nogaibay Hill (Sabieva and Kakimova, 2025). These examples demonstrate how pastoral practice and environmental description can remain legible in the lexicon of place. Dating remains the principal constraint because written attestation may postdate the naming event and a Turkic surface form may incorporate older substrate material. Anthropological-linguistic interpretation therefore requires attention to naming practice and cultural context (Völkel, 2023), while comparative research on South Siberian Turkic varieties illustrates the chronological complexity of language contact and shift (Novgorodov et al., 2015). Central Asian and Turkic studies of naming, cultural memory, and regional linguistic history provide additional context (Gaibullaev, 2023; Horsman, 2006; Nurdauletova et al., 2024; Kenbayeva et al., 2022; Bugybaykizi et al., 2015). These forms are not treated as a single synchronic substrate. Each attestation is assigned to the dated manuscript, political, and administrative phase in which it occurs, and a later Turkic surface form is not used by itself to date the origin of a name.

Figure 1 represents cross-script transmission as a staged evidentiary process. Solid arrows indicate recording or transmission from local forms into manuscript traditions and onward to source-critical comparison; dashed links indicate comparison among contemporaneous script traditions. The diagram does not assert direct equivalence among individual toponyms. Equivalence is assigned only after the provenance, phonological, chronological, and spatial controls described in Sections 2.6–2.7 and Section 8 have been evaluated.

**Figure 1:**
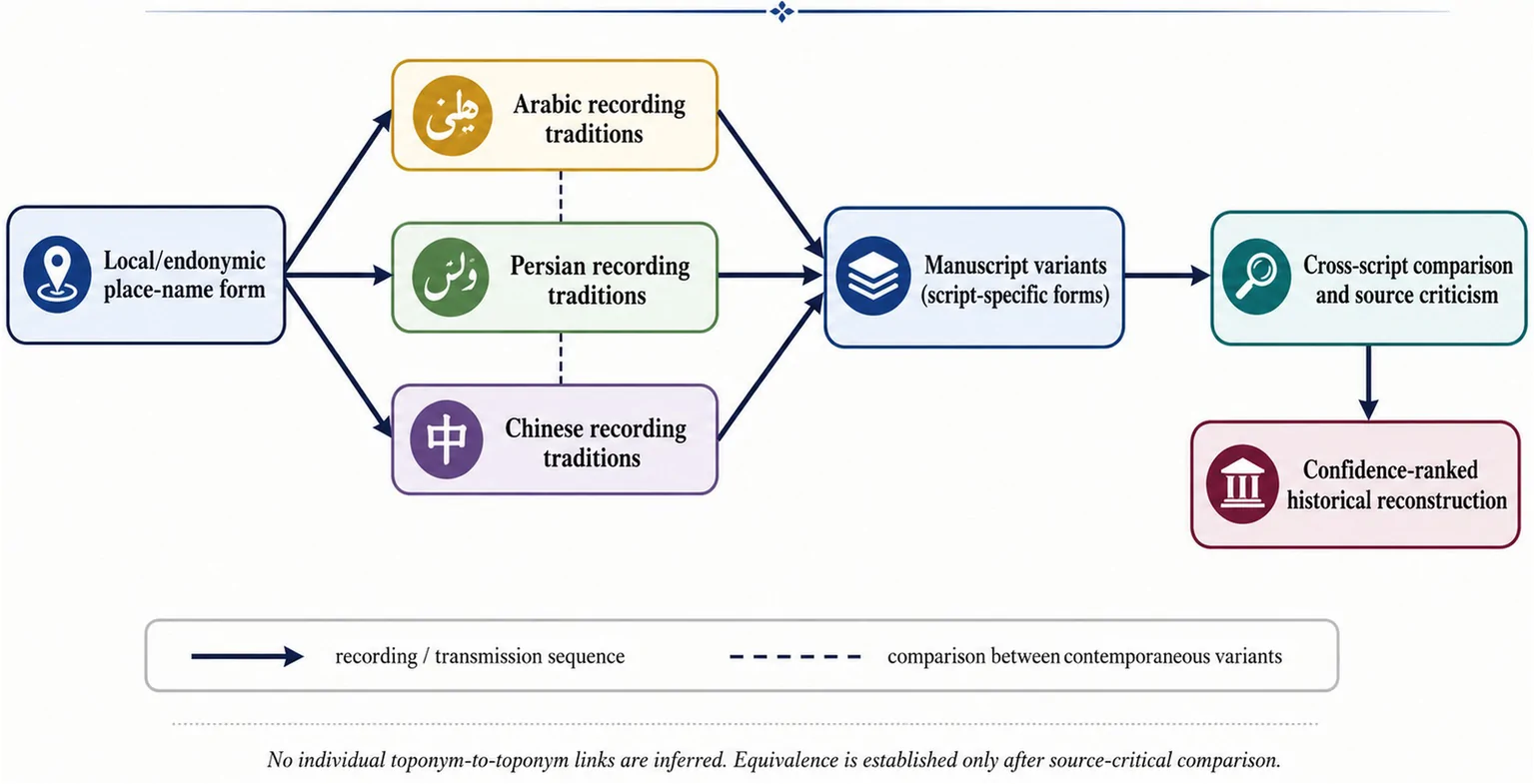
Cross-script transmission pathways for five-state Middle Asian toponyms (see Sections 3.1–3.3, 4.1–4.4, and 6.1).

### 4.2 Iranian and hybridised naming layers

Iranian and hybridised naming layers are especially important in the oasis and urban zones where Persian administrative and literary traditions interacted with Turkic-speaking populations. The Bukhara oasis provides a concrete example of why these layers cannot be separated by surface spelling alone: reconstructions of settlements behind Bukhara compare alternative manuscript forms with relative geography before accepting or rejecting proposed equivalences (Schwarz, 2022). Broader research on genetic and linguistic histories in Central Asia likewise demonstrates layered contact histories across populations and languages (Thouzeau et al., 2017). At the corpus-processing level, Persian named-entity recognition illustrates the technical difficulty created by variable orthography and contextual naming patterns (Ahmadi and Moradi, 2015). An Iranian or hybrid classification is therefore assigned only when linguistic form, manuscript context, and spatial evidence converge; ambiguous forms remain explicitly unresolved. Iranian and Persian scholarship on demographic history, ethnolinguistic layering, historical nomenclature, and toponym classification provides additional context (Vallini et al., 2024; Alizadeh, 2020; Mavaddat, 2025; Assadorian, 2017).

### 4.3 Multilingual Toponymic convergence zones

Multilingual convergence zones are most informative where several naming traditions refer to the same corridor, oasis, river, or settlement. Within the five-state core, Bukhara-area parallel forms demonstrate how manuscript variants must be compared against route sequence and relative geography (Schwarz, 2022). Kazakhstan provides a second type of evidence: geographical names undergo transliteration, phonetic adjustment, morphological modification, calquing, reinterpretation, and political renaming when they move across language and administrative systems (Zharkynbekova et al., 2024). On the eastern frontier, Xinjiang research documents the long-term evolution of a toponymic cultural landscape (Guo et al., 2025) and the geopolitical mobilisation of alternative toponyms in historical mapping (Hai, 2025). Xinjiang examples serve only as neighbouring controls for transmission mechanisms and are not counted as primary evidence for the five-state core.

Country-level evidence is not equally dense, so cross-country claims are calibrated to the sources available. In Kyrgyzstan, the capital’s documented sequence from Pishpek to Frunze and then Bishkek provides a direct case of politically mediated urban renaming (Thomas, 2025). In Tajikistan, the Pamir Mountains provide a direct case in which toponyms have been analysed as instruments linking landscape and ideology (Horsman, 2006). For Turkmenistan, historical-etymological analysis of 328 toponyms documented in Middle Eastern and East Asian sources provides direct evidence of cross-script variants and place-name evolution, including Dargan, Daukesken, Daya-Khatyn, and Koneurgenish (Abisheva et al., 2025). These cases complement the Kazakhstan and Bukhara evidence without assuming that all five states followed a single naming trajectory.

### 4.4 Diachronic Micro-cases and chronological sequencing

Historical layers are treated as temporally sequenced attestations rather than static language blocks. Each micro-case separates the observed written form, the language and script of the source, the plausible linguistic operation, and the historical inference that can safely be drawn. Phonetic transcription is supplied only where a source or a period-appropriate historical-phonological reconstruction supports the sound value; otherwise scholarly transliteration is retained to avoid false phonetic precision. This distinction is particularly important because a written resemblance can result from transcription practice rather than from direct linguistic descent (Schwarz, 2022; Zharkynbekova et al., 2024; Qiu et al., 2024). Table 2 provides the chronological scaffold for these comparisons; the micro-cases that follow test that scaffold against documented cross-script, morphological, semantic, and administrative transformations. Table 2 separates changes in recording authority from changes in spoken or inherited name forms. The sequence moves from external Chinese attestation, through Islamic-era Arabic geographical and Persianate administrative documentation, to late-medieval Turkic-Persianate re-documentation, imperial and Soviet standardisation and script reform, and post-Soviet endonym restoration. These phases are not treated as a single Iranian-to-Turkic substitution. Iranian-language oasis names, Turkic vernacular forms, Persianate bureaucratic spellings, and later state standards could overlap, be institutionally replaced, or survive in parallel; the evidentiary task is therefore to date each attestation and identify the authority that produced it before inferring linguistic succession.

**Table 2:** Diachronic framework for linguistic and administrative transformation of Middle Asian toponyms (see Sections 4.1–4.4, 6.1–6.3, and 8.1, 8.2).

Attestation/governance phase	Administrative and linguistic context	Toponymic process	Illustrative evidence and interpretive control
5th–7th centuries CE: post-Han Chinese external attestation	Sogdian/Iranian oasis polities and Chinese dynastic recording of western regions.	Character-based approximation of foreign names, exonym formation, and source-specific phonological adaptation; original pronunciation and first written attestation remain distinct.	Bukhara/Niumi provides a dated phonological control: Niumi is reported as Middle Chinese ṇjuk-mjet and Later Han Chinese ṇuk-mɨt, a close rendering of Sogdian Nōk-mētān. The identification is evaluated together with chronology and relative geography (Stark, 2021; Schwarz, 2022).
8th–13th centuries: Islamic geographical and Persianate administrative integration	Arabic geographical writing and Persian historiographical/administrative practice across Transoxiana, Bukhara and Khorezm.	Arabic-script variant formation, vowel underspecification, morphological variation, route-based stabilisation, and parallel exonyms/endonyms.	Dargan and the Jurjan/Jurjaniya/Gurganj variants are documented in Middle Eastern sources (Abisheva et al., 2025), while Bukhara source criticism shows that manuscript variants should be constrained by route position and relative geography (Schwarz, 2022).
14th–16th centuries: late medieval to early-modern re-documentation	Turkic-Persianate political succession, manuscript copying, and expanding interregional cartography.	Persistence of inherited names alongside cartographic abstraction, exonym use, and re-positioning of routes and rivers.	Jenkinson’s 1,562 map provides a dated cartographic checkpoint for Tashkent, Samarkand, the Oxus and Caspian relationships (Kollmann, 2024).
19th–20th centuries: imperial and Soviet standardisation	Centralised administration, script reform, official gazetteers and politically managed urban nomenclature.	Orthographic regularisation and deliberate replacement of names; political change can interrupt rather than phonologically transform a name lineage.	Pishpek to Frunze is treated as administrative renaming, not sound change (Thomas, 2025).
Post-Soviet period to present	National language policy, endonym restoration, multilingual official usage, transliteration reform and digital spatial standardisation.	Restoration or replacement of official forms, coexistence of legitimate variants, and renewed cross-script harmonisation requirements.	Frunze to Bishkek and contemporary Kazakh transliteration practices illustrate the distinction between renaming and orthographic adaptation (Thomas, 2025; Zharkynbekova et al., 2024).

Koneurgenish/Urgench provides the clearest cross-script micro-case in the reviewed five-state corpus. The Turkmenistan study records the Chinese forms Yue-gan/Yue-gyan alongside Khorezmian Gurganj and Arabic-source variants including Jurjaniya/Dzhurzhaniya, al-Gurganji, al-Jurjanii, and Gurganj. The same study reports the proposed internal development Gurganjird to Gurganj to Urgench and analyses the earlier form morphologically as Gurgan plus jird/gird/kird/kert, with the second element glossed as “city”. Crucially, it also cites Juvayni’s Persian-language Tarikh-i Jahangusha as preserving Jurjaniya and Urgench as alternative designations for the Khorezmian centre, providing an explicit Persian historiographical stage alongside the Chinese and Arabic evidence (Abisheva et al., 2025). This case therefore separates four analytically distinct operations: character-based phonetic approximation in the Chinese witness; Arabic-source exonymic and orthographic variation; Persian historiographical transmission of alternative designations; and a proposed morphological reduction within the Gurganj/Urgench lineage. The semantic proposal “city of Gurgan” is kept separate from the phonological comparison and is treated as a source-dependent etymology rather than as a demonstrated sound law. Because the cited Chinese and medieval written witnesses do not provide a period-secure IPA transcription for every stage, the review retains the scholarly transliterations actually reported by the source instead of assigning retrospective modern pronunciations; phonetic equivalence is tested only through the period-sensitive procedure specified in Section 8.2.

A second cross-script micro-case is provided by Bukhara. Source-critical scholarship treats the Chinese form Niumi together with the Numijkat/Nokmitan/Namit/Bukhara cluster as a diachronic identification problem rather than as a chain of visually similar spellings. The case is therefore used as a control on method rather than forced into a linear sound sequence: the Chinese transcription, Iranian-language forms, and later Islamic manuscript attestations are retained as separate evidence layers whose equivalence must be demonstrated through historical phonology, chronology, and relative geography. A published historical-phonological comparison makes the mechanism explicit: Niumi is reported as Middle Chinese ṇjuk-mjet and Later Han Chinese ṇuk-mɨt, values that have been compared with Sogdian Nōk-mētān (‘New Residence’). This provides an empirical example of period-specific segmental comparison rather than inference from modern Mandarin or graphic resemblance (Stark, 2021). A period-specific International Phonetic Alphabet transcription is not supplied for every remaining stage where the cited evidence does not provide a secure reconstruction; inventing one would convert orthographic evidence into unsupported phonetic certainty (Schwarz, 2022).

Dargan provides a morphological and semantic counter-case. Medieval Arabic geographical sources preserve Dargan on the Amu Darya route, while the modern place name Darganata/Dargan-Ata adds a later morphological element. The cited etymological study records competing explanations for the root Dargan, including an association with darga in the semantic field of boatman or ship captain (Abisheva et al., 2025). The referential continuity is therefore stronger than the semantic certainty: later morphological expansion can be described, but the root etymology remains a hypothesis rather than a settled reconstruction.

Aksu and Karasu illustrate semantic composition rather than cross-script phonetic evolution. The North Kazakhstan material analyses Aksu as ak, white, plus su, water, and places Karasu within the same regional hydronymic vocabulary (Sabieva and Kakimova, 2025). Their internal morphology can be evaluated independently of later orthographic conventions, so semantic transparency is coded separately from historical age. Bishkek provides a different mechanism again: the documented sequence Pishpek, Frunze, and Bishkek records politically mediated urban renaming (Thomas, 2025). These forms are not phonological stages of one name; their analytical value lies in dating changes in naming authority and official identity.

The chronology also avoids a simplistic model in which an Iranian layer is simply replaced by a Turkic one. In oasis settings, Iranian/Persian and Turkic naming practices overlap, and administrative language, manuscript language, and community usage can change at different rates (Schwarz, 2022; Thouzeau et al., 2017). Table 2 therefore presents phases of attestation and administrative mediation, not claimed dates of original name formation.

## 5 Manuscript cartography and spatial imagination

This section moves from manuscript text to cartographic form. Section 5.1 evaluates schematic and historical mapping as evidence for relative geography, and Section 5.2 examines the political authority embedded in the selection, prominence, and standardisation of map labels.

### 5.1 Schematic mapping traditions

Schematic manuscript maps communicate route hierarchy, regional order, and cultural geography through visual abstraction. Arabic-Islamic mapping traditions include both schematic and more mimetic forms, and their evidentiary value lies in the relationships they preserve among places, routes, seas, and regions (Pinto, 2016). For Central Asia, Anthony Jenkinson’s sixteenth-century cartographic scheme provides a concrete historical test case. Its representation of the Caspian region, the Oxus, Tashkent, and Samarkand can be compared with modern coordinates to identify changes in relative position and route geometry (Kollmann, 2024; Szykuła, 2012). Figure 2 uses four shared reference points in a historical-map panel and a modern coordinate panel. The comparison is qualitative; no numerical distortion index is calculated because the manuscript map and the coordinate map encode space according to different cartographic conventions.

**Figure 2:**
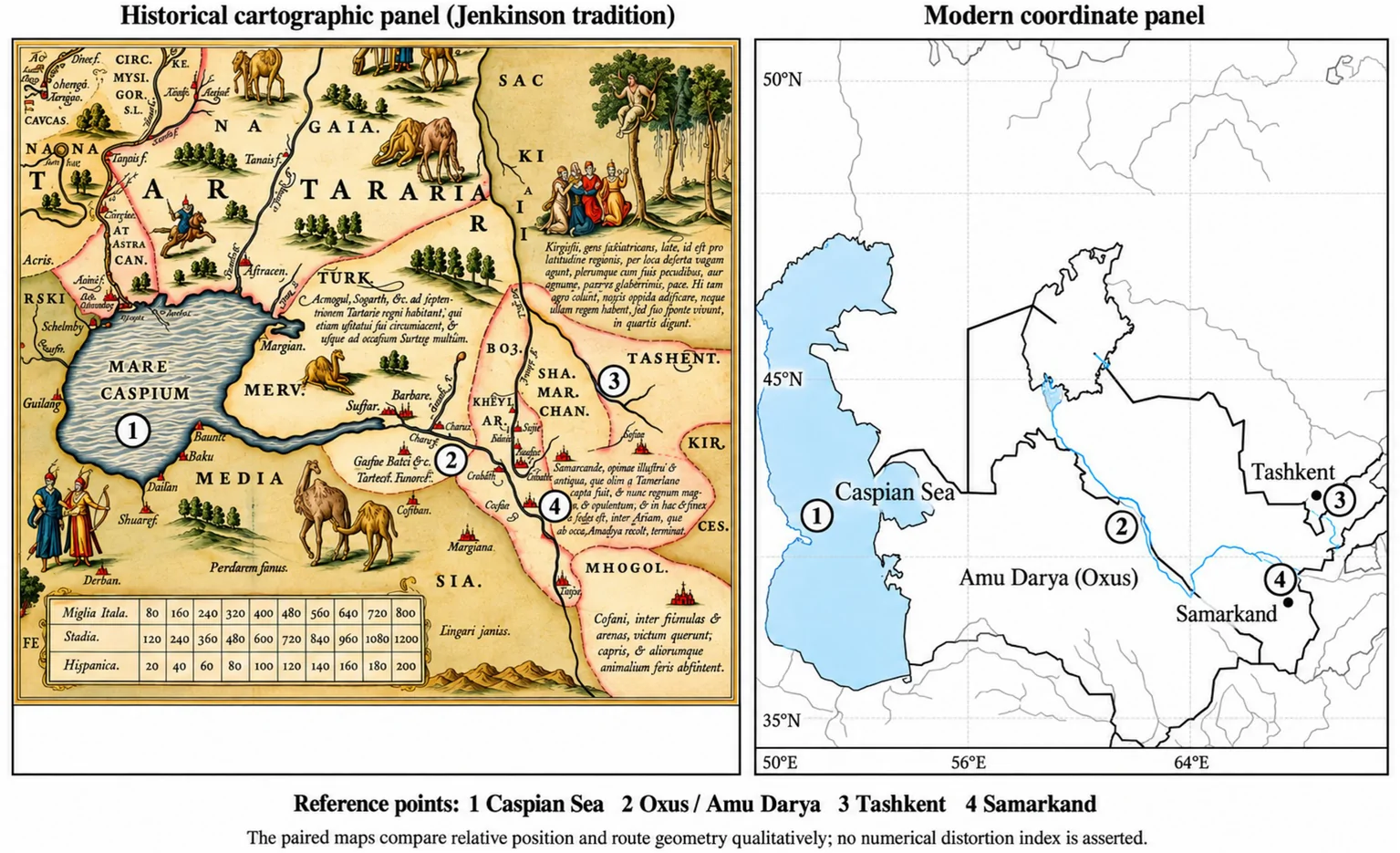
Historical-map and modern-coordinate comparison for selected Central Asian reference points. Reference points: 1 Caspian Sea; 2 Oxus/Amu Darya; 3 Tashkent; 4 Samarkand.

The paired-map design makes the comparison explicit. Reference point 1 marks the Caspian Sea, point 2 the Oxus/Amu Darya, point 3 Tashkent, and point 4 Samarkand. The purpose is to test relative spatial correspondence and displacement visually, not to derive a metric from a schematic historical representation.

### 5.2 Territorial visualisation and political authority

Historical cartography also records the politics of territorial visualisation. In Jenkinson’s Central Asian map, routes, regions, rivers, and selected urban names receive unequal visual prominence, and later revisions alter how the region is spatially organised (Kollmann, 2024). More generally, cartographic theory shows how maps participate in the production of territorial authority (Branch, 2011) and how official naming can privilege some spatial identities while marginalising others (Noucher, 2022). For toponymic reconstruction, the implication is practical: a label’s presence, absence, scale, or centrality is evidence about the institution that produced the map and cannot be read as a neutral measure of local importance.

## 6 Toponymic transformation mechanisms

This section separates three transformation mechanisms that require different reconstructive controls. Section 6.1 addresses phonetic distortion across scripts, Section 6.2 addresses semantic translation, and Section 6.3 addresses deliberate political renaming. The reconstruction-difficulty categories used in Table 3 are qualitative synthesis descriptors derived from the cited literature; they are not measured or statistically calibrated scores.

**Table 3:** Toponym transformation mechanisms across civilizations and recording systems (see Sections 4.1–4.4 and 6.1–6.3).

Mechanism	Linguistic driver	Political or administrative driver	Primary historical context	Typical manuscript or archival manifestation	Reconstruction difficulty	Dominant transformation domain	References
Phonetic transliteration distortion	Conversion between scripts with non-equivalent phonemes. Vowel omission or substitution.	Recording by administrators using dominant script system.	Multiscript environments such as Arabic, Cyrillic, and Chinese contexts.	Variant spellings across gazetteers and official registers.	High. Dependent on phonological back-reconstruction.	Linguistic	Zharkynbekova et al. (2024); Schwarz (2022)
Logographic approximation of foreign names	Representation of foreign sounds using logographic characters.	Bureaucratic incorporation of frontier regions.	Chinese dynastic gazetteer systems.	Character substitutions that approximate foreign pronunciation.	High. Phonetic recovery depends on historical phonology of characters.	Linguistic and administrative	Hung et al. (2008); Sproat and Gutkin (2021); Yokoi et al. (2024)
Political renaming after regime change	Replacement of earlier name with ideologically preferred designation.	Conquest. State consolidation. Nation building.	Transitional political systems.	Official decrees and updated administrative gazetteers.	Very high when earlier name is removed from official record.	Political	Bingol (2004); Zharkynbekova et al. (2024)
Administrative standardization	Orthographic normalization and elimination of variant forms.	Centralized census and taxation systems.	Imperial bureaucratic consolidation phases.	Uniform spelling in official gazetteers and fiscal records.	Moderate. Earlier forms often preserved in peripheral archives.	Administrative	Zharkynbekova et al. (2024)
Semantic calque translation	Translation of descriptive elements such as river, mountain, or new town into receiving language.	Administrative integration of multilingual territories.	Trade and frontier zones.	Parallel descriptive names in different languages.	Moderate. Comparative linguistic analysis can recover original meaning.	Linguistic	Sabieva and Kakimova (2025); Rojas-Garcia (2022)
Linguistic internal evolution	Gradual sound change within a single language across centuries.	Not primarily political. Occurs through natural linguistic development.	Long-settled regions with deep historical continuity.	Progressive phonetic shifts observable in manuscript strata.	High when early documentation is sparse.	Linguistic	Völkel (2023); Thouzeau et al. (2017)
Cartographic abstraction	Simplification of local name complexity in schematic maps.	Court-sponsored map production emphasizing territorial clarity.	Medieval atlas traditions and administrative mapping.	Shortened labels and generalized spatial ordering.	Moderate. Spatial relation preserved but phonetic nuance reduced.	Cartographic	Nazarkulova and Strobl (2015); Kollmann (2024)
Urban inscription reform	Systematic street and place naming for governance efficiency.	Municipal modernization and global integration.	Rapid urban expansion contexts.	Official address codification and map updating.	Low to moderate when documentation is preserved.	Administrative	Zharkynbekova et al. (2024)
Gazetteer compilation continuity	Repetition and updating of earlier place records in successive editions.	Institutionalized state record production.	Chinese dynastic and post-1949 gazetteer traditions.	Layered place entries with temporal annotation.	Low when archival continuity is strong.	Administrative	Du (2023); Murray (2021)
Spatial authority encoding	Emphasis on state territory and imperial hierarchy in map labeling.	Central political authority asserting legitimacy.	Song dynasty administrative mapping.	Hierarchical symbolization and central emphasis in maps.	Moderate. Spatial bias identifiable through contextual reading.	Cartographic and political	De Weerdt (2011); Gregory et al. (2018)
Exonym substitution	Adoption of externally used name in place of indigenous form.	Diplomatic practice. Imperial or colonial dominance.	Cross-cultural frontier regions.	Parallel naming traditions in external records.	High when indigenous form disappears from official documentation.	Linguistic and political	Jordan (2022); Zharkynbekova et al. (2024)
Endonym restoration	Reinstatement of indigenous names after regime transition.	Post-imperial or post-colonial identity recovery.	Political transition periods.	Legislative renaming acts and official gazette revisions.	Moderate. Earlier documentation often survives in archives.	Political	Bingol (2004); Zharkynbekova et al. (2024)
Script reform respelling	Systematic orthographic change following script transition.	State-driven language planning reforms.	Twentieth century script reforms in Eurasia.	Parallel Arabic, Latin, and Cyrillic spellings across archival layers.	High. Dependent on cross-script equivalence modelling.	Linguistic and administrative	Krouglov (2022); Oqlu (2021)
Toponymic layering	Coexistence of multiple historical names in parallel usage.	Multi-ethnic settlement without complete administrative erasure.	Long-term trade and migration corridors.	Simultaneous naming forms in multilingual gazetteers.	Very high. Historical-layer dating and attribution are required.	Linguistic and political	Giraut (2020)

### 6.1 Phonetic distortion

Phonetic distortion is a recurrent consequence of transferring Middle Asian names among Turkic and Iranian speech forms and Arabic, Persian, Chinese, Cyrillic, or Latin writing systems. The mechanism is structural: scripts differ in the contrasts they represent, and administrative standardisation may regularise variants that were previously unstable. Research on Kazakh geographical names documents transliteration, phonetic adjustment, morphological modification, lexical transformation, reduction, calquing, reinterpretation, and renaming across linguistic systems (Zharkynbekova et al., 2024). Bukhara-region source criticism provides a historical counterpart, showing that similar written forms cannot be equated until route position and neighbouring attestations have been checked (Schwarz, 2022). For each candidate reconstruction, this review therefore combines phonological plausibility, manuscript genealogy, relative geography, and independent historical context. Patterns of distortion identify the transmission pathway through which a name entered the archive. Central Asian reconstruction studies further demonstrate that cross-script comparison must remain historically constrained (Koshimova et al., 2026; Nurdauletova and Kenbayeva, 2026).

Figure 3 separates phonetic distortion, semantic translation, and political renaming because each mechanism carries a different reconstruction burden. Vertical arrows indicate the pathway from each mechanism toward manuscript-variant proliferation. Dashed outer boundaries represent uncertainty envelopes, and the reconstruction labels indicate confidence categories rather than deterministic outcomes.

**Figure 3:**
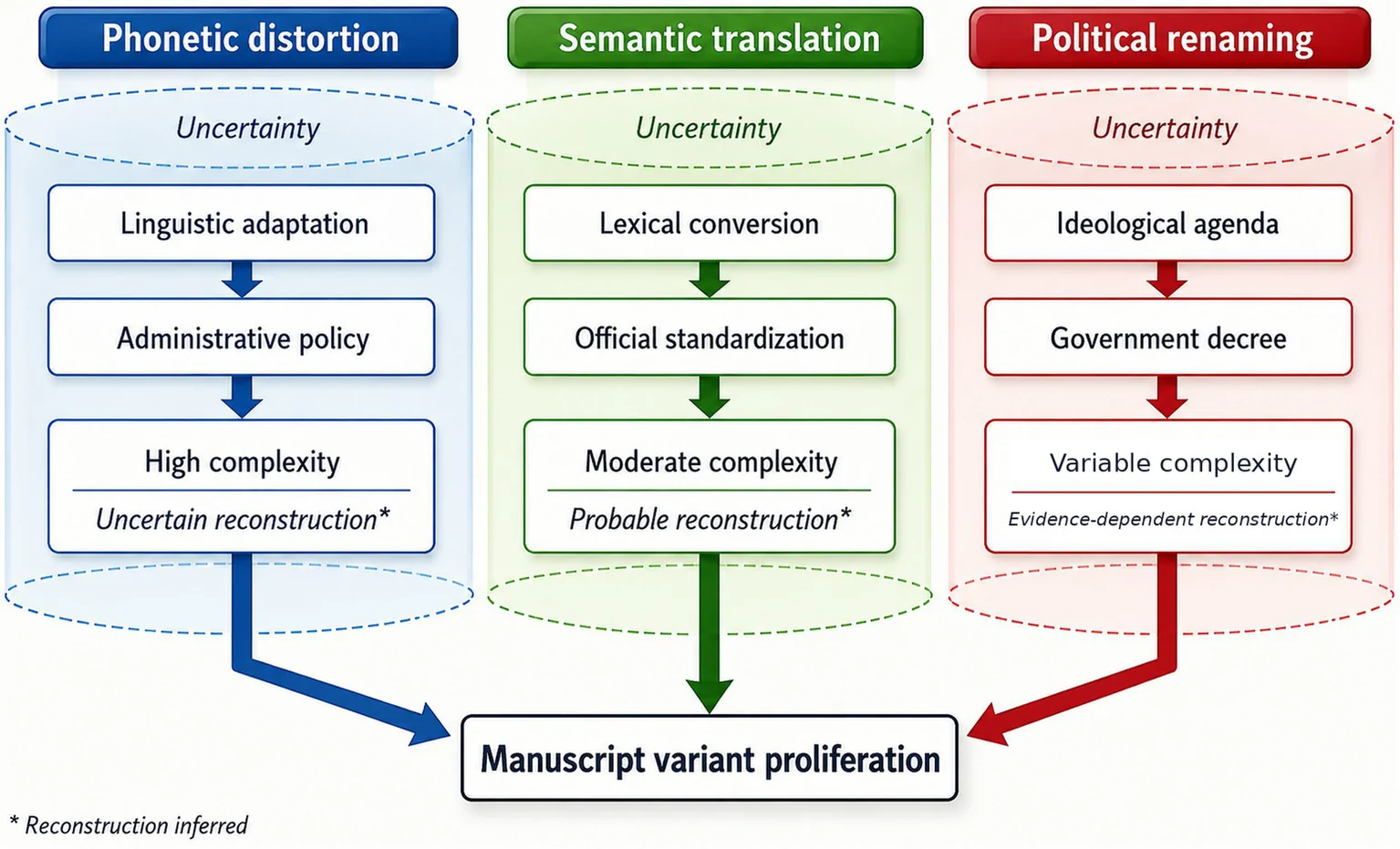
Toponym transformation mechanisms and reconstruction uncertainty (see Sections 6.1–6.3).

### 6.2 Semantic translation

Semantic translation transfers the descriptive content of a place name while changing its linguistic form. North Kazakhstan provides concrete examples of why local semantic systems matter. Aksu combines ak, ‘white’, with su, ‘water’, while Karasu forms part of a contrasting regional hydronymic vocabulary; interpretation depends on local associations with water source, flow, and landscape (Sabieva and Kakimova, 2025). River-name semantics more generally require explicit representation of the environmental and terminological context in which a hydronym is used (Rojas-Garcia, 2022). In the Pamir Mountains, context-specific ecological calendars further demonstrate how seasonal and environmental vocabulary is embedded in local livelihood knowledge (Ullmann et al., 2022). A semantic correspondence is therefore recorded separately from a phonetic correspondence, and a translated descriptor is not treated as proof that two forms share the same historical origin. Related work on Turkic ethnosemantics and semi-arid environmental interpretation provides complementary semantic controls (Konyratbayeva et al., 2024; Scholes, 2020).

### 6.3 Political renaming

Political renaming is an intentional transformation produced through official nomenclature, administrative reclassification, commemorative policy, or regime transition. Research on Kazakh geographical names explicitly identifies renaming and standardisation among the processes that alter place-name forms across administrative and linguistic systems (Zharkynbekova et al., 2024). Central Asian political transitions provide the broader context in which state-building and nationalisation can reshape public nomenclature (Bingol, 2004). Comparative critical-toponymy research shows that regime collapse, decolonisation, and government change frequently trigger systematic place renaming (Lim and Perono Cacciafoco, 2021). Such changes are historically informative because the decree, gazetteer revision, or administrative act can often be dated; the principal reconstructive risk is the disappearance of older local forms from official records. Critical-toponymy and sacred-geography studies provide additional controls on how naming authority becomes institutionalised (Alderman, 2022; Tsyrempilov et al., 2021; Kadmon, 2004; Wanjiru-Mwita and Giraut, 2020).

Figure 4 organises reconstruction as a sequential evidence-evaluation pathway. It does not assume that naming and reconstruction form a self-repeating historical cycle. New evidence can require an interpretation to be revisited, but that is a normal consequence of source criticism and does not constitute a cyclic model of toponymic change.

**Figure 4:**
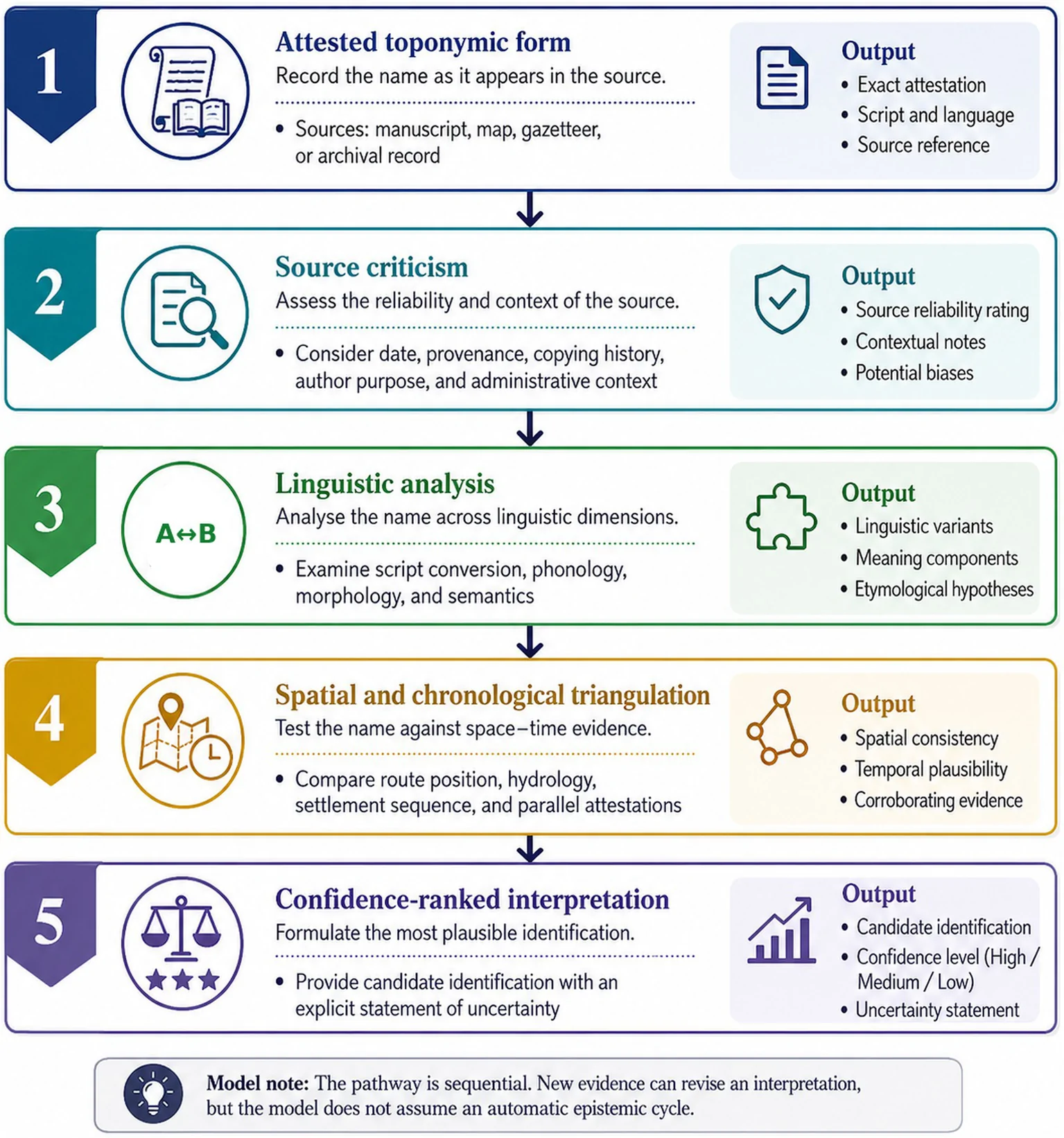
Sequential evidence-evaluation pathway for five-state Middle Asian toponymic reconstruction (see Sections 6 and 8).

## 7 Ethnogenesis and Toponymic evidence

### 7.1 Toponymic persistence and mobility

Toponymic persistence can reveal the geographic memory of movement and affiliation when names recur across settlements, pastures, routes, and oral traditions. Nogai place names demonstrate how ethnocultural memory can remain distributed across maps, archives, and migration histories (Bulgarova, 2023). North Kazakhstan adds more spatially specific examples, including ethnonym-bearing names such as Kyrghyz upland and Nogaibay Hill and settlement terminology derived from seasonal pastoral practice (Sabieva and Kakimova, 2025). Long-run evidence on nomadic corridors across Asian highlands provides an independent mobility context for interpreting such distributions (Paik and Shahi, 2023). These associations support hypotheses about contact, movement, and remembered affiliation; they do not establish a direct one-to-one correspondence between a historical name and a modern ethnic population. Regional studies of identity and ethnocultural memory provide complementary evidence for interpreting persistence without equating it with origin (Yama and Wasino, 2025; Meirbekov and Meiirbekov, 2021).

### 7.2 Hydronyms, Oronyms, and environmental memory

Hydronyms and oronyms often have high persistence because rivers, springs, mountains, passes, and pasture zones remain practical reference points across political change. North Kazakhstan hydronyms such as Aksu and Karasu preserve environmental descriptors within a Turkic lexical system (Sabieva and Kakimova, 2025). In the Manas River Basin, water-related settlement names show a measurable relationship between toponymic heritage, hydrological setting, and settlement distribution (Zhang et al., 2025). In the Pamir Mountains, ecological calendars demonstrate the context-specific links among seasonal language, environmental observation, and livelihood practice (Ullmann et al., 2022). Environmental names therefore provide strong evidence for landscape use and local classification when their historical meaning is triangulated with hydrology, topography, and settlement chronology. Kazakhstan-focused studies of landscape terminology, speleonyms, hydronyms, and colour symbolism provide additional environmental context (Bazarbekov et al., 2024; Amirbekova et al., 2025; Dautkulova et al., 2026; Khassenov et al., 2022).

### 7.3 Limits of Ethnogenetic inference

Ethnogenetic interpretation requires a stricter evidentiary threshold than lexical or spatial description. Naming practices are socially embedded and may survive changes in language, population, or political authority (Völkel, 2023). Historical place-name scholarship shows that official renaming and inherited naming can coexist across political transitions, while cartographic standards influence which parallel names become publicly visible (Williamson, 2023; Mitxelena-Hoyos and Amaro-Mellado, 2025). In Kazakhstan, contemporary transliteration and translation practices further demonstrate that one geographical referent can acquire several legitimate forms under different linguistic and administrative rules (Zharkynbekova et al., 2024). The analysis therefore distinguishes persistence from origin, distribution from identity, and official visibility from community usage. Ethnogenesis is inferred only when toponymic evidence converges with independent historical, archaeological, linguistic, or demographic evidence.

## 8 Methodological bottlenecks in Toponymic reconstruction

Reconstructive accuracy is constrained by three linked bottlenecks. Chronostratigraphic attribution separates the naming event from surviving written attestations; cross-script harmonisation relates script-specific forms to plausible spoken originals; and cartographic-textual integration tests whether a linguistic identification is spatially credible. Sections 8.1–8.3 treat these problems separately so that uncertainty can be traced to its source.

### 8.1 Chronostratigraphic attribution

Chronostratigraphic attribution distinguishes the date of a naming event, the date of first surviving attestation, the date of later copying, and the date of administrative standardisation. Kazakhstan evidence shows why semantic form alone cannot establish age: ancient Turkic toponyms may preserve older cultural and environmental information even when surviving documentation is later (Imanberdiyeva et al., 2025). In the Bukhara oasis, manuscript variants and relative geography must be evaluated together before a historical settlement is identified (Schwarz, 2022). Historical cartographic revision provides an additional chronological control because later maps can preserve inherited spatial schemes alongside new observations (Kollmann, 2024). Reliable attribution therefore combines manuscript genealogy, archaeological occupation, administrative history, route context, and environmental continuity. The preferred result is a bounded date range or confidence-ranked phase, not an unsupported single origin date.

### 8.2 Cross-script harmonisation

Cross-script harmonisation reconstructs plausible relationships among written forms produced by different orthographic systems. Arabic and Persian-script evidence is treated first as an orthographic record rather than as a complete phonetic transcription: short-vowel information may be absent from ordinary Arabic and Persian writing, so written consonantal evidence must be kept distinct from reconstructed vocalism (Saiegh-Haddad and Henkin-Roitfarb, 2014; Nayernia et al., 2019). Chinese characters can approximate foreign syllables through historically variable pronunciations, and later Latin or Cyrillic standards can regularise earlier variants. Kazakhstan research documents the practical consequences of transliteration and translation for geographical names (Zharkynbekova et al., 2024). Chinese toponym matching and historical named-entity recognition provide computational methods for comparing large variant sets (Qiu et al., 2024; Liu et al., 2021), while writing-system research clarifies why phonographic recovery from logographic representation is structurally uncertain (Sproat and Gutkin, 2021). Each harmonised candidate is accompanied by the observed variants, proposed sound correspondences, provenance, and a confidence level. Comparative work on phonetic transfer across scripts provides an additional control (Sobakina and Semenova, 2025).

For reproducibility, candidate equivalence is evaluated through an ordered harmonisation protocol. First, the source graphemes, source language, manuscript date, and attested spelling are preserved unchanged, with any modernised spelling stored separately. Second, the transliteration printed in the critical edition or source-critical study is retained as the primary Romanised form; any additional normalised Romanisation used for digital comparison is stored separately and never replaces the attested spelling. Transliteration is never treated as a phonetic reconstruction, and IPA is supplied only where a modern pronunciation or a period-specific historical reconstruction is independently supported. Third, for Arabic- and Persian-script forms, the written consonantal frame is evaluated separately from any short vowels absent from the attested form, and alternative vowel restorations remain explicit hypotheses unless a critical edition or independent source-specific reconstruction resolves them (Saiegh-Haddad and Henkin-Roitfarb, 2014; Nayernia et al., 2019). Fourth, Chinese character transcriptions are compared only against period-appropriate historical reconstructions or source-critical readings. Modern Mandarin values are not substituted for earlier pronunciations, and modern character readings are never back-projected as historical evidence (Baxter and Sagart, 2014). Computational matching studies are used only for candidate generation and variant alignment (Qiu et al., 2024; Liu et al., 2021), not as authorities for historical Chinese phonology. Writing-system evidence is then used only to characterise the structural limits of logographic-to-phonographic recovery (Sproat and Gutkin, 2021). Fifth, each candidate is segmented into roots, affixes, titles, and generic geographical elements before whole-form similarity is judged. Sixth, proposed correspondences such as velar/uvular alternation, voicing adjustment, vowel epenthesis, or syllable restructuring are accepted only when they are compatible with the phonotactics and documented adaptation practices of the relevant languages and period; no universal substitution rule is imposed across unrelated source traditions (Zharkynbekova et al., 2024). Seventh, a historical equivalence is accepted only when phonological plausibility is supported by chronology and at least one independent spatial or documentary control. A chronological or geographic contradiction is treated as disqualifying unless a demonstrable copying, exonymic, or transmission pathway explains it. The output therefore reports graphemic similarity, phonological plausibility, morphological compatibility, semantic compatibility, spatial-chronological corroboration, and residual uncertainty as separate evidentiary dimensions. No candidate is accepted on orthographic resemblance alone: at minimum, a proposed equivalence must show phonological plausibility, chronological compatibility, and either spatial or independent documentary corroboration; otherwise it remains an unresolved variant or low-confidence hypothesis.

A proposed cross-script correspondence is accepted only when five conditions converge: the consonantal correspondences are compatible with the source and receiving systems; vowel differences can be explained by documented orthographic underspecification or historically plausible adaptation; any syllable restructuring is compatible with the receiving language or script; morphological segmentation yields recurrent and independently interpretable elements rather than *ad hoc* fragments; and the direction of change is chronologically possible. A match that depends on unexplained metathesis, deletion, substitution, or retrospective reading from a modern spelling is rejected or assigned lower confidence. These criteria separate transliteration, phonetic approximation, morphological change, and semantic translation instead of treating overall visual resemblance as a sound law.

The Koneurgenish micro-case illustrates the protocol. Yue-gan and Gurganj-related forms are not collapsed because they look approximately similar in romanisation; the comparison first preserves their independent source traditions, then asks whether period-specific sound reconstruction, route position, and historical sequence can support a common referent (Abisheva et al., 2025). Where one of these controls remains unresolved, the correspondence remains a ranked hypothesis rather than a normalised canonical form.

### 8.3 Cartographic-textual integration gaps

The third bottleneck is the separation of philological reconstruction from spatial testing. A proposed linguistic match remains weak if the corresponding site is incompatible with route sequence, hydrology, travel distance, or neighbouring settlements. Jenkinson’s Central Asian map illustrates how the relative placement of the Caspian, Oxus, Tashkent, and Samarkand changes across cartographic schemes (Kollmann, 2024). Regional digital-atlas work demonstrates the feasibility of shared geospatial infrastructure for Central Asia (Nazarkulova and Strobl, 2015), while toponym-assisted georeferencing and temporal graph matching provide reproducible methods for aligning historical labels with modern spatial entities (Bahgat and Runfola, 2021; Khokhlova et al., 2022). An integrated workflow can reject spatially impossible equivalences, identify plausible missing waystations, and distinguish copied labels from geographically grounded attestations. Further cartographic and spatial-method evidence is synthesised in Table 4 (Mitxelena-Hoyos and Amaro-Mellado, 2024; Muroň et al., 2024; Puerta et al., 2025; Huang et al., 2025; Piškinaitė and Veteikis, 2023; Aitken, 2024).

**Table 4:** Synthesis of evidentiary functions and reconstruction bottlenecks in Sections 7 and 8.

Analytical issue	Evidence in five-state Middle Asia	Main inference	Principal uncertainty	Section
Ethnonym-bearing settlement and orographic names	North Kazakhstan ethnonym-bearing forms and Nogai naming memory	Contact, mobility, and remembered affiliation	Names can outlive language shift or population movement	7.1
Hydronyms, oronyms, and environmental names	Aksu/Karasu patterns; Manas Basin water-related settlement names; Pamir ecological vocabulary	Environmental memory, resource use, and route continuity	Meanings can be reinterpreted across periods and languages	7.2
Chronostratigraphic attribution	Name origin, manuscript attestation, copying, and later standardisation	Temporal layering of the toponymic record	First surviving attestation is not equivalent to name origin	8.1
Cross-script harmonisation	Arabic/Persian vowel underspecification; Chinese rendering; later Latin/Cyrillic forms	Candidate equivalence across scripts	Historical phonology and orthographic ambiguity	8.2
Cartographic-textual integration	Historical-map labels, route sequences, hydrology, and modern coordinates	Spatial plausibility of a proposed identification	Copied, displaced, or selectively omitted labels	8.3

## 9 Digital and computational Toponymics

Digital and computational methods address the three bottlenecks identified in Section 8 when provenance and uncertainty remain visible throughout the workflow. The infrastructure considered here has four components: GIS-linked manuscript mapping, cross-script and phonological matching, route-network modelling, and interoperable historical gazetteers.

### 9.1 GIS-linked manuscript mapping

GIS-linked manuscript mapping aligns historical maps, itinerary descriptions, manuscript place lists, and archaeological coordinates within a common spatial framework. Central Asian cartographic history supplies direct material for this task, including Jenkinson’s revised sixteenth-century representation of the region (Kollmann, 2024) and regional atlas prototyping that demonstrates the feasibility of shared geospatial infrastructure (Nazarkulova and Strobl, 2015). Toponym-assisted georeferencing can use historical names as anchors when the matching procedure records ambiguity and uncertainty (Bahgat and Runfola, 2021), while graph-based geographic matching can link spatial entities whose boundaries or labels change through time (Khokhlova et al., 2022). For the five-state core, the resulting map becomes a test environment in which manuscript identifications can be checked against terrain, hydrology, route distance, and known settlement sequences.

### 9.2 Cross-script and phonological matching

Cross-script matching compares candidate variants while retaining the historical rules that produced them. Kazakhstan provides a direct empirical basis because geographical names exhibit transliteration, phonetic adjustment, calquing, reinterpretation, and administrative renaming across languages and scripts (Zharkynbekova et al., 2024). Chinese toponym-matching models and historical named-entity recognition show how large corpora can be searched for variant geographical entities (Qiu et al., 2024; Liu et al., 2021), and Persian named-entity recognition supplies a complementary approach for Persian textual material (Ahmadi and Moradi, 2015). The appropriate output is a ranked set of candidate correspondences with explicit phonological steps and provenance, allowing a specialist to accept, reject, or refine the automated match. Automated string similarity is therefore used only to generate candidates. Confirmation requires the same source-period phonology, morphological segmentation, chronology, geography, and provenance tests specified in Section 8.2, so computational matching cannot substitute surface orthographic resemblance for historical equivalence.

### 9.3 Route-network modelling

Route-network modelling treats toponyms as nodes in historically constrained mobility systems. Evidence for long-run nomadic corridors across Asian highlands provides a regional basis for connecting mobility and route structure (Paik and Shahi, 2023), while Silk Road network reconstruction shows how dispersed heritage nodes can be analysed as a connected spatial system (Shen et al., 2024). Multi-criteria historical-route simulation provides a transferable modelling technique for testing environmental constraints and alternative paths (Litvine et al., 2024). Applied to Kazakhstan, Kyrgyzstan, Tajikistan, Turkmenistan, and Uzbekistan, such models can compare proposed manuscript routes with seasonally feasible travel corridors, known caravan nodes, topography, and water availability. Their strongest use is hypothesis testing: an implausible route weakens a proposed identification, whereas a plausible route identifies where further textual or archaeological evidence should be sought. Regional trade and mobility modelling provides complementary methods for testing route significance (Wang et al., 2022; Liu, 2024).

### 9.4 Interoperable historical gazetteers

Interoperable historical gazetteers provide the data layer that connects variant names to stable spatial entities without erasing their differences. Global linguistic datasets demonstrate the value of standard identifiers and georeferenced language distributions (Ranacher et al., 2025). Methods for reconciling cartographic and toponymic databases show how redundancies and variant labels can be detected in multilingual settings (Mitxelena-Hoyos and Amaro-Mellado, 2023), while linked-open-data approaches connect topographic features to formal geographic knowledge structures (Usery et al., 2018). Historical travel-record extraction adds a textual pathway for discovering and relating place entities across sources (Ghosh and Chatterjee, 2025). A five-state Middle Asian gazetteer built on these principles would preserve each attested form, script, date, source, proposed correspondence, coordinate estimate, and confidence value as separate but linked records. Computational studies of neural decoding, digital toponymy, Chinese spatial data, and cross-language database design provide complementary infrastructure models (Wang et al., 2025; Serikova and Baishukurova, 2022; Liu et al., 2024a; Law et al., 2025).

Together, these infrastructures make uncertainty explicit and evidence interoperable. GIS tests spatial plausibility; script-aware matching compares variants at scale; network models evaluate route feasibility; and linked gazetteers preserve provenance across attestations. Their analytical value depends on transparent assumptions, versioned data, confidence scoring, and continued philological review. Figure 5 summarises this architecture by separating evidence inputs, normalisation and alignment, analytical operations, and research outputs.

**Figure 5:**
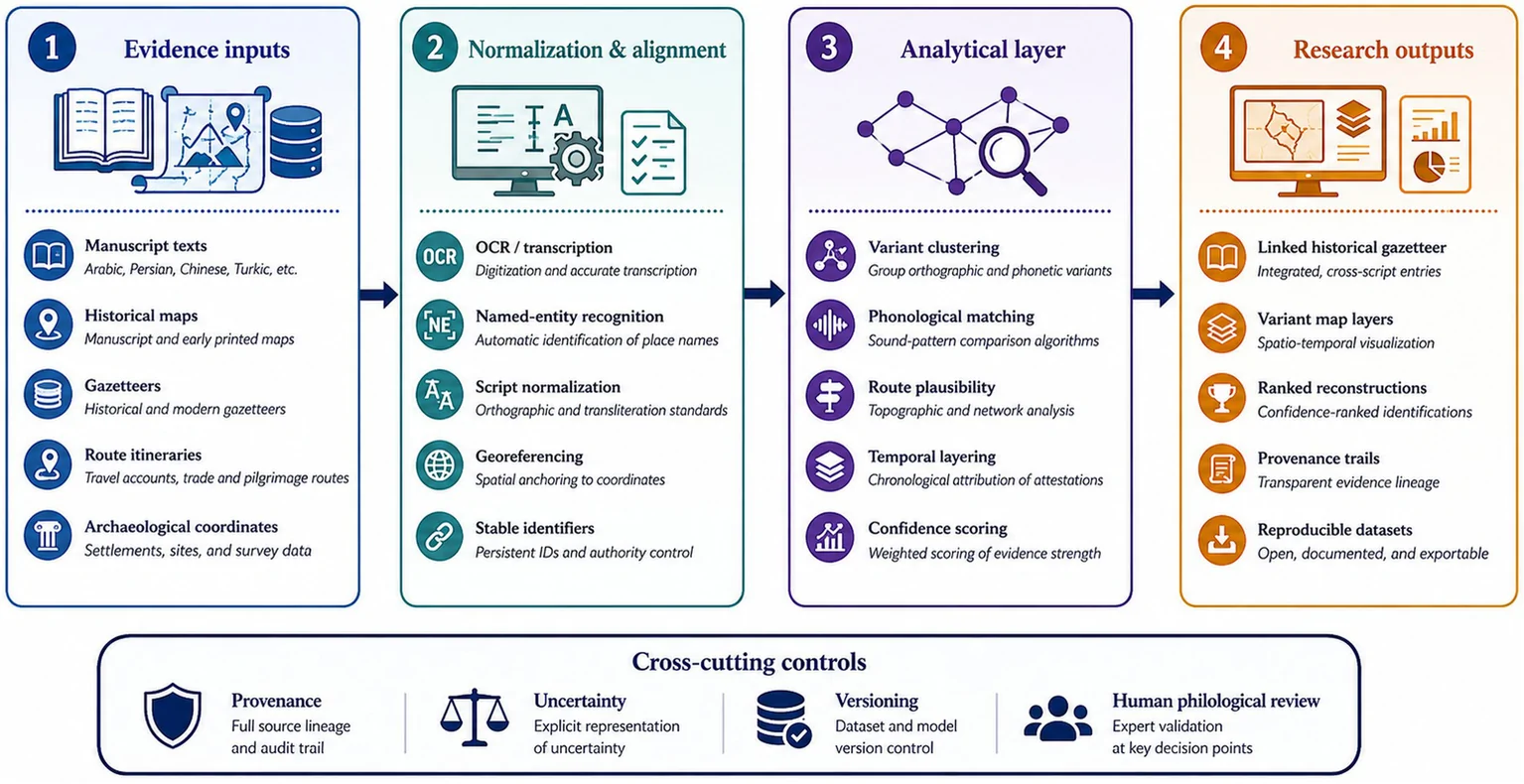
Digital and computational toponymics architecture for five-state Middle Asia (see Sections 9.1–9.4).

## 10 Conclusion

The evidence synthesised in this review supports a more precise interpretation of Middle Asian toponymy as a layered historical archive. Arabic, Persian, and Chinese recording traditions preserved different aspects of the same regional geography, while Turkic, Iranian, and hybrid naming layers recorded mobility, oasis administration, environmental knowledge, and political change within Kazakhstan, Kyrgyzstan, Tajikistan, Turkmenistan, and Uzbekistan. The clearest regional cases show that a historical form becomes analytically useful when its linguistic structure, manuscript provenance, relative location, and chronological position are considered together. The principal analytical lesson is that toponymic reconstruction depends on triangulation. Name origin must be separated from first surviving attestation; persistence must be separated from population identity; and official standardisation must be separated from community usage. These distinctions turn apparent variant spellings into testable historical observations and place explicit limits on ethnogenetic or territorial claims. The same framework also clarifies the role of neighbouring evidence: Tarim Basin, Xinjiang, Chinese, Iranian, Russian, Afghan, and wider Eurasian materials are most informative when they document transmission pathways, comparative mechanisms, or boundary mediation connected to the five-state core. The next research phase should prioritise interoperable primary-source infrastructure for the region: digitised manuscript attestations with stable provenance, a trans-script historical gazetteer, coordinate-linked route and settlement records, and confidence-ranked procedures for cross-script matching. Progress also depends on publishing unresolved variants rather than forcing premature harmonisation, linking philological interpretation to archaeological and spatial evidence, and building country-connected corpora that can be updated as new sources are edited or georeferenced. These steps would move the field from dispersed inventories toward reproducible historical-geographical reconstruction while preserving the linguistic and manuscript complexity that gives Middle Asian toponymy its evidentiary value. The diachronic analysis sharpens the theoretical contribution. Toponymy qualifies as a knowledge infrastructure when a name is repeatedly used to index and connect spatial records under identifiable institutional conditions, not merely because the name survives in a text. The micro-cases also show why transformation mechanisms must remain analytically separate: Koneurgenish/Gurganj is primarily a cross-script equivalence problem, Dargan/Dargan-Ata combines referential continuity with morphological and semantic uncertainty, Aksu/Karasu illustrate semantic composition, and Pishpek-Frunze-Bishkek represents administrative replacement rather than regular sound change. This mechanism-specific separation provides a reproducible basis for historical-linguistic and GIS integration without flattening chronological, phonological, or political differences.
